# Feasibility and diagnostic accuracy of fast whole-body MRI in slightly to moderately injured trauma patients

**DOI:** 10.1007/s00330-024-10933-y

**Published:** 2024-07-12

**Authors:** Katrin Reichel, Patricia Hahlbohm, Marie-Luise Kromrey, Heiner Nebelung, Felix Schön, Konrad Kamin, Jens Goronzy, Jens-Peter Kühn, Ralf-Thorsten Hoffmann, Sophia Freya Ulrike Blum

**Affiliations:** 1 Institute and Polyclinic for Diagnostic and Interventional Radiology, University Hospital Carl Gustav Carus, Technical University Dresden, Fetscherstraße 74, 01307 Dresden, Germany; 2 University Center of Orthopaedic, Trauma and Plastic Surgery, University Hospital, TU Dresden, Fetscherstr. 74, 01307 Dresden, Germany

**Keywords:** Whole body imaging, Feasibility studies, Magnetic resonance imaging, Trauma centers, Advanced trauma life support care

## Abstract

**Objectives:**

To determine the feasibility and diagnostic accuracy of fast whole-body magnetic resonance imaging (WB-MRI) compared to whole-body computed tomography (WB-CT) in detecting injuries of slightly to moderately injured trauma patients.

**Materials and methods:**

In a prospective single-center approach, trauma patients from convenience sampling with an expected Abbreviated Injury Scale (AIS) score ≤ 3 at admission, received an indicated contrast-enhanced WB-CT (reference standard) and a plain WB-MRI (index test) voluntarily up to five days after trauma. Two radiologists, blinded to the WB-CT findings, evaluated the absence or presence of injuries with WB-MRI in four body regions: head, torso, axial skeleton, and upper extremity. Diagnostic accuracy was determined using sensitivity, specificity, positive predictive value, and negative predictive value by body region.

**Results:**

Between June 2019 and July 2021, 40 patients were assessed for eligibility of whom 35 (median age (interquartile range): 50 (32.5) years; 26 men) received WB-MRI. Of 140 body regions (35 patients × 4 regions), 31 true positive, 6 false positive, 94 true negative, and 9 false negative findings were documented with WB-MRI. Thus, plain WB-MRI achieved a total sensitivity of 77.5% (95%-confidence interval (CI): (61.6–89.2%)), specificity of 94% (95%-CI: (87.4–97.8%)), and diagnostic accuracy of 89.3% (95%-CI: (82.9–93.9%)). Across the four regions sensitivity and specificity varied: head (66.7%/93.1%), torso (62.5%/96.3%), axial skeleton (91.3%/75%), upper extremity (33.3%/100%). Both radiologists showed substantial agreement on the WB-MRI reading (Cohen’s Kappa: 0.66, 95%-CI: (0.51–0.81)).

**Conclusion:**

Regarding injury detection, WB-MRI is feasible in slightly to moderately injured trauma patients, especially in the axial skeleton.

**Clinical relevance statement:**

Besides offering a radiation-free approach, whole-body MRI detects injuries almost identically to whole-body CT in slightly to moderately injured trauma patients, who comprise a relevant share of all trauma patients.

**Key Points:**

- *Whole-body MRI could offer radiation-free injury detection in slightly to moderately injured trauma patients*.
- *Whole-body MRI detected injuries almost identically compared to whole-body CT in this population*.
- *Whole-body MRI could be a radiation-free approach for slightly to moderately injured young trauma patients*.

## Introduction

Traumatic injuries are a leading cause of death in individuals aged 5–29 years [1]. Annually, Europe witnesses approximately 36 million injured patients, with 5% requiring inpatient care and 0.6% succumbing to traumatic injuries [2]. Detecting the relatively small amount of severely injured patients, defined by an injury severity score (ISS) over 15 [3], upon their hospital arrival remains a tremendous challenge. This difficulty results in over-triage rates of up to 87% [4, 5]. The standard for diagnosing injuries of severely injured trauma patients is whole-body computed tomography (WB-CT) [6]. WB-CT is crucial for the survival of severely injured trauma patients enabling a timely diagnosis and treatment of life-threatening injuries [7]. However, the indication for WB-CT is made by the trauma team after clinical evaluation and assumption of severe injury leading to an error-prone decision-making to justify WB-CT [6]. In addition, the lowest adherence to guidelines in trauma care can be found in computed tomography (CT) imaging [8].

WB-CT exposes patients to high radiation doses of approximately 20 millisieverts (mSv) representing a diagnostic procedure with one of the highest radiation exposures [9–11]. The risk of radiation-induced neoplasia increases in younger individuals and risk assessments suggest that 1:500 trauma patients aged < 20 years might succumb due to the radiation exposure of one WB-CT [12]. Based on the current overall mortality rate of 5% per Sievert [13], a 20 mSv WB-CT has a mortality rate of 1 in 1000. Therefore, a routine WB-CT for suspected severely injured trauma patients to rule out life-threatening injuries must be viewed critically. This is particularly important in younger patients, who represent a significant share of trauma patients. For example, 17% of all trauma patients in the United Kingdom were younger than 25 years in 2013 [14].

To address this issue, current research determined whether WB-CT or a selective CT strategy decreases trauma mortality showing no difference [15]. However, even a selective CT approach often results in consecutive scans of all body parts indicating a benefit of initial whole-body imaging [15]. Solving the issue by using whole-body magnetic resonance imaging (WB-MRI) as a radiation-free and contrast agent-free approach has only been shown feasible in pediatric trauma patients [16, 17].

We aimed to evaluate the feasibility and diagnostic accuracy of WB-MRI compared to routine WB-MRI for injury detection in slightly to moderately injured trauma patients. Furthermore, radiation dose estimations were obtained on each WB-CT [9–11].

## Methods

The study was conducted in accordance with the principles of the Declaration of Helsinki and Good Clinical Practice guidelines. Furthermore, ethical approval was obtained from an independent local ethics committee. Written informed consent was obtained from all patients before participation.

### Participants

This prospective single-center study was conducted between June 2019 and July 2021 at a level 1 trauma center that provides trauma care for an area of 7.944 km^2^ and a population of 1.6 million inhabitants [18]. Trauma patients admitted to the emergency department receiving a WB-CT were included through convenience sampling. The inclusion criteria were: age of 18 years or older, absence of magnetic resonance (MRI) contraindications, an Abbreviated Injury Scale (AIS) ≤ 3 [19] in all body regions estimated by a senior trauma surgeon (K.K.) before WB-CT, and written informed consent. Accidents associated with suicide or crime, immediate surgical treatment, and secondary patient transfer, lead to exclusion. A WB-MRI was performed within five days after WB-CT. Mechanism of injury, gender, age, height, weight, ISS [3], duration of WB-MRI, and time between WB-CT and WB-MRI were recorded. Furthermore, the radiation exposure of each WB-CT was estimated by using individual dose-length products. Besides the convenience series, the total number of WB-CT examinations during the study period at our trauma center was assessed for over-triage calculation. Over-triage was defined by the share of WB-CT scans in trauma patients with ISS ≤ 15 [3, 20]. Injuries were classified as clinically relevant by either a prolonged hospital stay or the necessity for surgical intervention.

The results of the indicated WB-CT were used as the reference standard, whereas the voluntary WB-MRI was seen as the index test.

### Trauma management and reference standard

The trauma team managed trauma patients according to the advanced trauma life support and determined the need for WB-CT consistent with the guidelines of the European Society of Emergency Radiology [6, 21]. Scans were performed using a Siemens Somatom Definition Edge 2 × 64-slice spiral CT scanner (Siemens Healthineers) located adjacent to the resuscitation area. First, a native scan of the head was acquired, followed by a contrast-enhanced CT-angiography (aortic valve to vertex) after administration of 80 milliliters of Ultravist 370 (Bayer Vital GmbH) contrast agent. Finally, a 65-second post-contrast scan of the thorax, abdomen, and pelvis was obtained.

### Index test

**Fig. 1:**
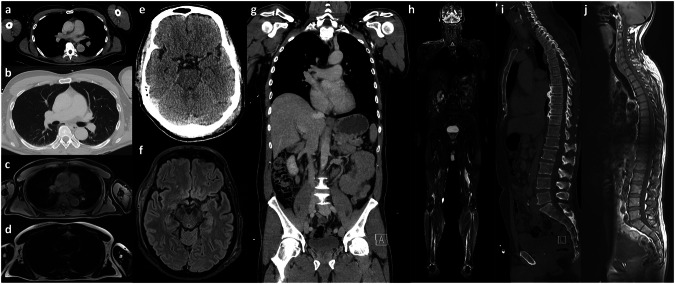
Example of whole-body imaging in CT and MRI. A 59-year-old patient who had a bike accident not wearing a helmet. Transverse CT slices of the thorax in soft tissue window (**a**) and lung window (**b**) and corresponding MRI in DIXON sequence with (**c**) and without fat saturation (**d**). Transverse CT-slices of the brain in soft tissue window (**e**) and corresponding FLAIR-sequence (**f**), both depicting a temporal galea hematoma on the right side and a temporooccipital galea hematoma on the left side. Coronal reconstruction of the venous phase of thorax and abdomen in soft tissue window (**g**) and composed coronal TIRM-sequences of the whole patient (**h**). Sagittal CT reconstruction of the spine in the bone window (**i**) and composed sagittal T1-weighted MRI images (**j**)

Each participant undergoing WB-MRI was examined in a supine position using body coils in a 3.0 Tesla Siemens Magnetom Prisma (Siemens Healthineers). No contrast agent injection was used. The total estimated scanning time was 16:04 min. Table 1 shows the protocol of the WB-MRI, after a scout of 37 s. Figure 1 shows examples of the standard scans of the WB-MRI protocol compared to WB-CT.

**Table 1:** Scanning protocol of the fast whole-body MRI

Sequence	Body regions	Echo time (ET) in milliseconds	Repetition time (TR) in milliseconds	Slice thickness/gap (millimeter) matrix size	Duration (minutes)
Turbo inversion recovery magnitude	Thorax, upper extremity, abdomen, pelvis, lower extremity (coronal)	53	5010	5/6 256 × 256	9:20
T1 turbo spin echo	Spine (sagittal)	8.4	661	3/3.3 640 × 640	3:08
Fluid-attenuated inversion recovery	Brain (axial)	85	9000	3/3.3 320 × 320	2:44
T1-Dixon with breath-hold	Lung (axial)	1.34	4.21	3 320 × 320	0:15

### Reading of CT and MRI

Estimations of the diagnostic accuracy were performed by evaluating the presence of injuries within four predetermined body regions: head, torso, axial skeleton, and upper extremity.

Reading of the WB-CT was performed by a senior radiologist on call immediately after the presentation to the trauma team on a workstation with two high-resolution monitors using the local PACS software (IMPAX EE R20 XIX SU1, Agfa HealthCare N.V.). Final reports were used for aggregating injuries within one body region to a negative or positive finding within the predetermined four body regions.

Three months after completion of the last WB-MRI, two board-certified senior radiologists (S.F.U.B. 10-year experience, J.P.K. 18-year experience) independently reviewed each WB-MRI examination. The readers were unaware of the clinical background and injury extent found in WB-CT and evaluated the existence of injuries in the analogous predetermined four body regions as either positive or negative following a dichotomous decision tool. Differences in interpretation between the readers were resolved by consensus reading. Finally, reading results of WB-CT and WB-MRI were compared and all mismatches were followed by a detailed evaluation of WB-CT and WB-MRI by both readers determining the injury leading to a positive finding in the body region. False positive body regions in WB-MRI were interpreted as CT-occult findings.

### Statistical analysis

Normal distribution of all data was tested using Kolmogorov-Smirnov test. Mean and standard deviation were reported for normally distributed variables, otherwise, median, and interquartile ranges (IQR) were used.

Cohen’s Kappa (κ) was determined by evaluating the interreader agreement according to the classification of Landis and Koch: almost perfect (1 ≥ κ ≥ 0.8), substantial (0.8 ≥ κ ≥ 0.6), moderate (0.6 ≥ κ ≥ 0.4), slight (0.4 ≥ κ ≥ 0.2), or poor (0.2 ≥ κ ≥ 0) [22].

The diagnostic accuracy of WB-MRI was assessed with sensitivity, specificity, positive predictive value (PPV), negative predictive value (NPV) including 95% confidence intervals (95%-CI), and area under the receiver operating characteristic (ROC) curves for all body regions.

### Dose estimation

Estimates of the effective dose in mSv of each WB-CT were calculated by multiplying body-region-specific dose-length-product with organ-specific conversion factors of the American Association of Physicists in Medicine [23].

## Results

**Fig. 2:**
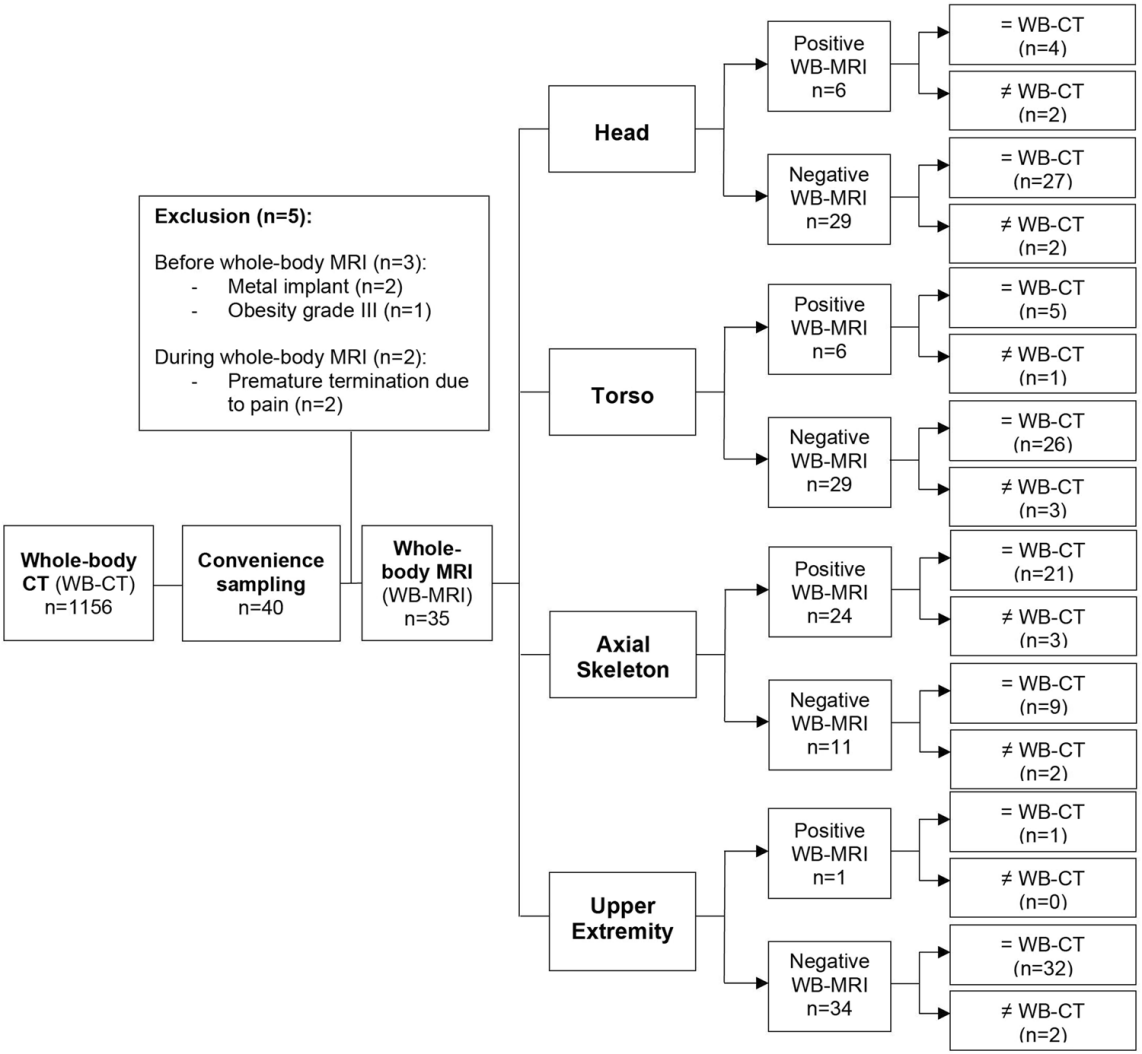
Flow of participants

During the study period, 1156 trauma patients received a WB-CT. Among them, 475 patients showed an ISS ≤ 15 accounting for an over-triage of 41%. Out of the 40 patients forming the convenience sample, 5 were excluded as shown in Fig. 2. Thus, 35 patients (median age 50 years (IQR: 32.5), 26 male) completed WB-MRI and were included in the study. Demographics and baseline clinical characteristics are presented in Table 2.

**Table 2:** Demographics and baseline characteristics

*n*	35
age in years – median (Interquartile range (IQR))	50 (32.5)
‐range, years	18–73
male gender % (*n*)	74.3% (26)
Body-mass-index in kg/m^2^ – mean (IQR)	24.9 (3.4)
Mechanism of injury in % (*n*)	
‐car accident	29% (10)
‐bike accident	23% (8)
‐motorbike accident	23% (8)
‐pedestrian	3% (1)
‐fall > 3 m	11% (4)
‐fall < 3 m	11% (4)
Injury severity score (ISS) – mean (IQR)	6 (8.5)
‐range	1–27
‐ISS > 15 in % (*n*)	8.6% (3)

The median estimated effective dose of the WB-CT was 18.9 mSv (IQR: 6.7 mSv) with a minimum of 12.6 mSv and a maximum of 33.3 mSv.

None of the patients received any clinical interventions between WB-CT and WB-MRI. The median time difference between WB-CT and WB-MRI was 47 h (IQR: 32 h). The median actual scanning time of WB-MRI was 24:20 min (IQR: 5:42 min). No adverse events occurred during WB-MRI.

For WB-MRI, overall interrater reliability was κ = 0.66 (95%-CI: (0.51–0.81)), equaling a substantial agreement.

A total of 140 body regions (35 patients × 4 regions) were assessed as shown in Table 3. In 125 of 140 body regions, the findings of the WB-CT and WB-MRI were consistent. Overall, the diagnostic accuracy of WB-MRI was 89.3% (95%-CI: (82.9–93.9%)). The overall sensitivity was 77.5% (95%-CI: (61.6–89.2%)), and specificity 94% (95%-CI: (87.4–97.8%)). An overall PPV of 83.8% (95%-CI: (70–92%)) and a NPV of 91.3% (95%-CI: (85.4–94.9%)) were obtained. Sensitivity and specificity by body region are shown in Table 4.

**Table 3:** Cross-tabulation of the whole-body MRI (index test) and whole-body CT (reference standard)

	Whole-body CT		
Whole-body MRI	Positive	Negative	Total
Positive	31	6	37
Negative	9	94	103
Total	40	100	140

**Table 4:** Sensitivity and specificity by body region

Region	Sensitivity (95%-CI)	Specificity (95%-CI)
Head	66.7% (22.3–95.7%)	93.1% (77.2–99.2%)
Torso	62.5% (24.5–91.5%)	96.3% (81–99.9%)
Axial Skeleton	91.3% (72–98.9%)	75% (42.8–94.5%)
Upper Extremity	33.3% (0.8–90.6%)	100% (89.1–100%)
Whole-body	77.5% (61.6–89.2%)	94% (87.4–97.8%)

**Fig. 3:**
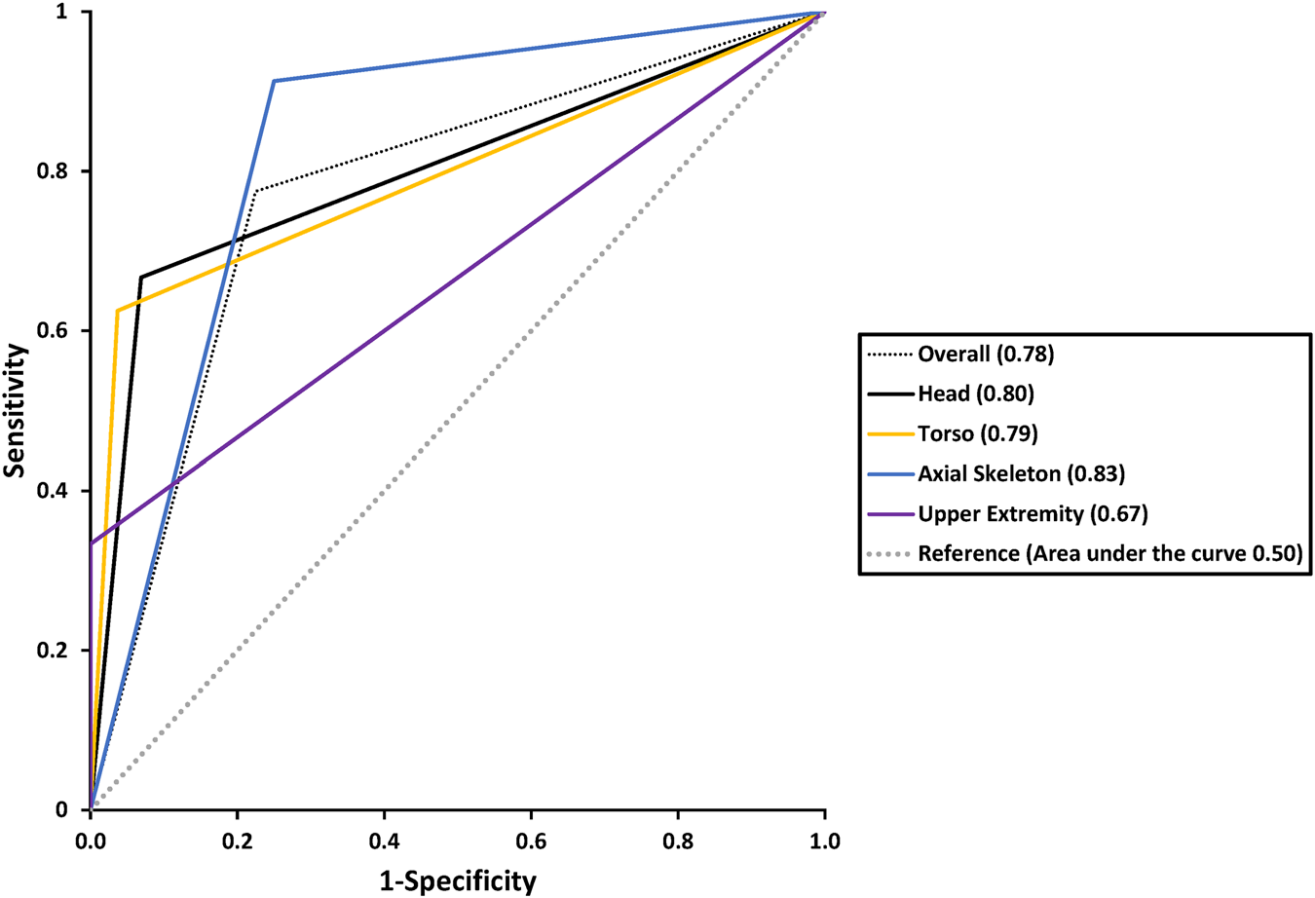
Receiver operating characteristics (ROC) curve for each body region and whole body

Figure 3 summarizes the diagnostic accuracy of the predetermined body regions with ROC curves. The highest area under the curve was seen in the axial skeleton (0.83) and lowest in the upper extremity (0.67). All missed injuries including their clinical relevance are shown in Table 5. Four missed injuries in WB-MRI prolonged the hospital stay, of which three required surgical intervention and one solely clinical observation. Therefore, four out of nine missed injuries were clinically relevant (44%). No missed injury was life-threatening.

**Table 5:** Missed injuries in whole-body MRI and clinical relevance

Injury type	Injury occurrence	Abbreviated injury scale (AIS)	Clinical relevance
Subdural hematoma	1	3	24-hour monitoring
Midfacial fracture (zygomatic arch and lateral orbital wall)	1	3	Surgical treatment of zygomatic arch fracture
Minimal lung contusion	3	2	Respiratory therapy
Rib fracture	1	1	None
Manubrium fracture	1	2	None
Distal radius fracture	2	2	Surgical treatment

WB-MRI identified false positive findings in 6 body regions, which comprised of one artifact and 5 CT-occult findings: a galea hematoma, a buccal soft tissue edema, a presacral hematoma, a clavicula contusion, and a rib contusion.

**Fig. 4:**
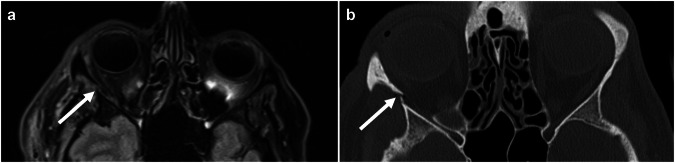
Axial plane of a fracture of the right lateral orbital wall (white arrows). **a** Fluid attenuated inversion recovery (FLAIR) image (axial MRI section; 3 mm thick) depicting the slightly dislocated fracture. No associated hematoma is found. **b** CT of the same patient showing the same fracture morphology

**Fig. 5:**
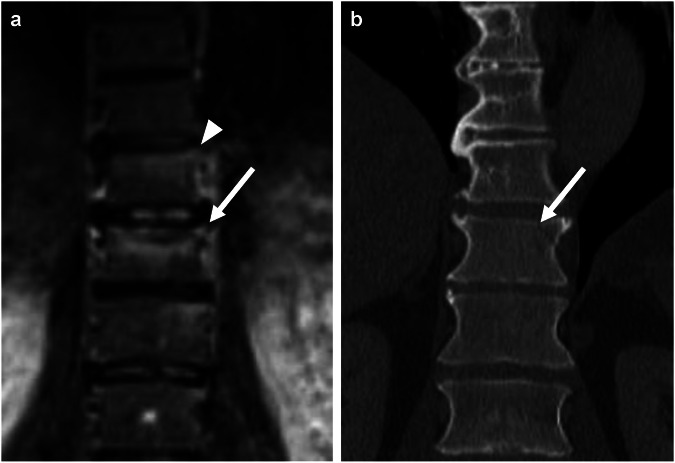
Coronary plane of a fracture of the 11th thoracic vertebra (white arrows). **a** Turbo Inversion Recovery Magnitude (TIRM) image (coronal MRI, 5 mm thick) showing a hyperintense line underneath the superior vertebral endplate and a hyperintensity underneath the superior vertebral endplates of the 10th and 12th thoracic vertebra, corresponding to bone bruise (white arrowhead). **b** CT of the same patient only depicted the fracture of the 11th thoracic vertebra

Figure 4 shows an example of a false negative finding in WB-MRI: a fracture of the orbital wall as a component of a missed midfacial fracture not having been identified with WB-MRI. Conversely, Fig. 5 shows the correct identification of a spinal fracture in WB-MRI with enhanced detectability through findings in the vertebrae above and below.

## Discussion

Addressing the inevitable high rates of over-triage in trauma care, we aimed to investigate whether WB-MRI could identify injuries of slightly to moderately injured trauma patients comparably. Our results suggest that a WB-MRI could detect injuries of slightly to moderately injured trauma patients comparably to WB-CT with minor limitations. All WB-CT scans were associated with a notable median radiation dose equivalent to approximately nine years of natural radiation exposure in Germany [24].

The outstanding sensitivity of WB-CT ranging from 89.6–98.5% in injury detection should be considered within the context of severely injured patients with an ISS > 15 [25–27]. While Smith et al ascertained a sensitivity of 98.5% in severe combat trauma, Yoong et al assessed a sensitivity of 98% for solely major trauma [26, 27]. Stengel et al determined that WB-CT detects injuries in severely injured patients with an ISS > 15 with a sensitivity of 89.6%. However, despite a high rate of over-triage, only 37% of the study population were categorized as severely injured. The sensitivity decreased to 77.2% in the larger portion with an ISS ≤ 15 (63%), a detail not explicitly mentioned by Stengel et al [25]. Most likely sensitivity decreases with lower injury severity. Therefore, the sensitivity of WB-MRI observed by our study might reflect the sensitivity of whole-body imaging in slightly to moderately injured patients that is lower in general. WB-MRI’s limitation in detecting upper extremity injuries may result from incomplete imaging of the arms since they were positioned next to the body and therefore not fully captured. Even WB-CT is limited in detecting fractures of the hand and forearm when not the entire arm is included in the scan, comparable to the results of our study of around 40–45.5% [28]. Questioning their clinical relevance, minor lung contusions that accounted for most missed injuries might not have been diagnosed before the implementation of WB-CT in trauma care [29]. Whole-body imaging in trauma patients is intended to identify life-threatening injuries, which none of all missed injuries in WB-MRI aligned with.

As mortality from radiation exposure increases and mortality from slight to moderate traumatic injuries decreases in younger trauma patients, the need for protection from the long-term radiation risks associated with WB-CT becomes relevant. Laack et al reported a mortality risk of 0.6% for intermediate trauma with a median ISS of 8. This risk was six times higher than the long-term risk of radiation-induced neoplasia by a single WB-CT. However, the authors state that no trauma-related death occurred in patients under 80 years and all trauma-related deaths were observed in patients with a median age of 90 years in a study population of 642 patients with a mean age of 43.8 years [12]. According to Baker et al, trauma patients over 70 years of age with an ISS of 10–19 showed eight times higher death rates than those aged younger than 50 years [3]. Conversely, one WB-CT scan increases the estimated long-term mortality from radiation exposure fourfold from 0.05% (> 60 years) to 0.2% (< 20 years) in younger patients [12].

WB-MRI is effective for detecting axial skeleton injuries, especially in the spinal region. Pizones et al determined a 91% sensitivity and 100% specificity in detecting injuries of the spinal posterior ligamentous complex of the thoracolumbar spine using MRI [30]. Although our current protocol could not offer the quality for evaluating disco-ligamentous injuries, further enhancements in the imaging sequences could enable immediate assessment of spinal stability in spinal trauma cases. Besides, MR shows higher sensitivity in diagnosing injuries of the spinal cord and acute pelvic fractures [31, 32]. Especially in pregnant trauma patients, it could detect placental abruptions with higher certainty [33]. MRI offers superior soft tissue contrast, potentially enhancing the detection of life-threatening injuries such as bleeding and organ contusions with higher certainty. However, no such events were observed in our study. Nevertheless, as more patients are included in future research, the likelihood of identifying such occurrences may increase, potentially revealing WB-MRI as a superior method for detecting life-threatening injuries.

Longer acquisition time using MRI might limit the feasibility in the acute trauma setting. Johnson et al showed that MRI acquisition time could be halved using deep learning reconstruction while maintaining the equivalent quality of the original protocol in knee MRI [34]. Therefore, scanning time is anticipated to be reduced in the future. Furthermore, the first whole-body CT presented by Leidner et al had a median examination time of 20 min [35]. Integrating WB-MRI into standard trauma management would require the availability of MRI facilities and the continuous presence of radiologists who are familiar with trauma WB-MRI interpretation. Hence, an important limitation of MRI in the trauma setting is the unknown existence of metal implants which imply an MRI contraindication.

### Limitations

Our study had several limitations. First, its generalizability is limited by data collection at a single center, a small number of participants, convenience sampling, and voluntary participation. Second, the interpretation was limited to injury detection and aggregation of injuries within one area. Furthermore, using WB-CT as a reference standard limits the diagnostic accuracy of WB-MRI since the procedure itself shows no level 1 evidence for diagnosing trauma patients [36]. Dose estimations are constrained by the utilization of general conversion factors, not including patient-specific characteristics such as weight in the calculation. Hence, the diagnostic accuracy of WB-MRI is limited to the whole-body protocol used in this study and might vary by change of sequences or readers. Furthermore, our protocol represents only one possibility of obtaining a WB-MRI showing the various options of sequence composition when using MRI instead of CT for whole-body imaging. Three-dimensional isotropic T1-weighted sequences or short tau inversion recovery imaging could have been included and represent options for potential optimization of the protocol. Sensitivity for injuries of the head might improve by including susceptibility weighted imaging sequences in the protocol. The lower resolution of MRI could limit the ability to diagnose discrete bone lesions, for example in the midface. Furthermore, the ability to detect injuries in WB-MRI could have been comprised by the delay of up to five days between WB-CT and WB-MRI. Minor lung contusions, the most frequently overlooked injuries in our study, exhibit radiographic variations as early as 6 h following thoracic trauma and usually heal within 3–5 days [37].

## Conclusion

Given the high rates of over-triage, a significant proportion of slightly to moderately injured trauma patients undergo WB-CT. For young patients who have a higher risk of developing radiation-induced neoplasia, whole-body MRI could detect injuries almost identically to WB-CT offering a radiation-free approach.

## Abbreviations

CI: Confidence interval
CT: Computed tomography
IQR: Interquartile range
ISS: Injury severity score
MRI: Magnetic resonance imaging
mSv: Millisievert
NPV: Negative predictive value
PPV: Positive predictive value
ROC: Receiver operating characteristic
WB-CT: Whole-body computed tomography
WB-MRI: Whole-body magnetic resonance imaging
κ: Cohen´s Kappa
